# Clinical features and management of postoperative lumbar intervertebral space infections following spinal endoscopy: a retrospective analysis

**DOI:** 10.3389/fcimb.2025.1538779

**Published:** 2025-05-19

**Authors:** Kunpeng Su, Mingzhi Liu, Mengxuan Wang, Qingyu Yao, Zirui Wang, Zheng Lian, Chuanli Zhou

**Affiliations:** ^1^ Department of Spinal Surgery, The Affiliated Hospital of Qingdao University, Qingdao, Shandong, China; ^2^ Department of Medicine, Qingdao University, Qingdao, Shandong, China

**Keywords:** spinal endoscopy, intervertebral space infections, surgical site infection, infection prevention, retrospective study

## Abstract

**Background:**

Postoperative lumbar intervertebral space infections following spinal endoscopy are infrequent but severe complications that can markedly affect patient recovery and treatment outcomes. Early diagnosis remains challenging due to the nonspecific nature of clinical symptoms. This study aims to identify the clinical characteristics, risk factors, and effective diagnostic and treatment strategies for postoperative intervertebral space infections.

**Methods:**

A retrospective analysis was conducted on 14 cases of postoperative intervertebral space infections following spinal endoscopy. The data set included patient demographics, clinical symptoms, imaging findings, blood culture results, and treatment approaches. The analysis assessed early diagnosis and treatment outcomes concerning the infection’s progression and the use of MRI, inflammatory markers, and empirical antibiotics.

**Results:**

The most common clinical symptoms included localized back pain, neurological deficits, and fever, though these lacked specificity. MRI proved valuable in diagnosing early infections. The majority of cases exhibited elevated levels of inflammatory markers, such as CRP and ESR. The treatment plan included early surgical intervention with debridement and internal fixation, along with extended antibiotic therapy.

**Conclusion:**

The early identification and intervention of postoperative intervertebral space infections following spinal endoscopy are of critical importance. The implementation of a strict aseptic technique, the execution of careful preoperative planning, and the timely use of MRI for diagnosis are essential to the prevention and effective treatment of these infections. This study underscores the necessity of a comprehensive approach to minimize the risk of postoperative intervertebral space infections and to enhance patient outcomes.

## 1 Introduction

Degenerative spinal diseases, including disc herniation and spinal stenosis, represent a significant global health concern, affecting over 540 million people annually and imposing a considerable socioeconomic burden. These conditions are a leading cause of disability worldwide, underscoring the urgent need for effective prevention and treatment strategies (Hoy et al., 2012; James et al., 2018). Although traditional open procedures, such as spinal fusion, are efficacious treatments, their extensive tissue damage and elevated infection risks frequently result in patient reluctance (Zaina et al., 2016; Crocker et al., 2021). As a representative of advanced minimally invasive techniques, endoscopic spine surgery is becoming increasingly favored by both patients and surgeons (Yue and Long, 2015; Kanno et al., 2019). Over the past three decades, advancements in endoscopic techniques and instrumentation have enabled surgeons to achieve comparable or superior clinical outcomes through spinal endoscopy surgery while minimizing tissue disruption, reducing operative blood loss, and shortening recovery times (Han et al., 2022). These benefits have rendered spinal endoscopy surgery a representative approach for conditions such as disc herniations, spinal stenosis, and infections (Verdú-López et al., 2014; Choi et al., 2016).

Despite the reduced incidence of postoperative infections in endoscopic procedures compared to open spinal surgeries, these complications still represent a significant challenge in clinical practice (Mueller et al., 2019; Lin et al., 2023). Surgical site infections have the potential to result in prolonged hospital stays, the necessity for repeat interventions, and even permanent neurological deficits (Dowdell et al., 2018; Lener et al., 2018). Historical data show that minimally invasive techniques, particularly spinal endoscopy, reduce the risk of infection. The incidence of surgical site infections following traditional open spinal surgeries ranges from 1.9% to 16% (Mueller et al., 2019; Freire-Archer et al., 2023; Pivazyan et al., 2023). In contrast, the rates of infection in endoscopic spine surgery are markedly lower, as evidenced by multiple cohort and case-control studies (Ee et al., 2014; Kulkarni et al., 2016; Mueller et al., 2019).

The postoperative infection rate is influenced by several key factors, including the patient’s comorbidities, the duration of the surgical procedure, and adherence to sterile protocols (Meng et al., 2015). Among these, diabetes and obesity are consistently identified as significant predictors of infection risk due to their impact on wound healing and immune response (Mehta et al., 2013; Martin et al., 2016). The integration of perioperative optimization strategies, such as glycemic control and weight management, highlights the necessity of a multidisciplinary approach to spinal surgery.

Although the benefits of spinal endoscopy in reducing infections are well documented, postoperative monitoring and early intervention remain of paramount importance. The prompt identification of infection through clinical and laboratory evaluations, followed by targeted antimicrobial therapy, can often mitigate severe complications (Lener et al., 2018; Pivazyan et al., 2023). Despite the lower infection rates observed in spinal endoscopic procedures compared to open spinal surgeries, postoperative infections remain a significant challenge that necessitates a multidisciplinary approach to management.

This paper aims to present a comprehensive review of the postoperative infection risks associated with spinal endoscopy, with a particular emphasis on evidence-based perioperative management strategies. By consolidating findings from a range of clinical studies and case series, this work seeks to highlight the critical role of infection prevention in maximizing the potential of minimally invasive spinal surgery.

## 2 Methods

### 2.1 Patients

**Figure 1:**
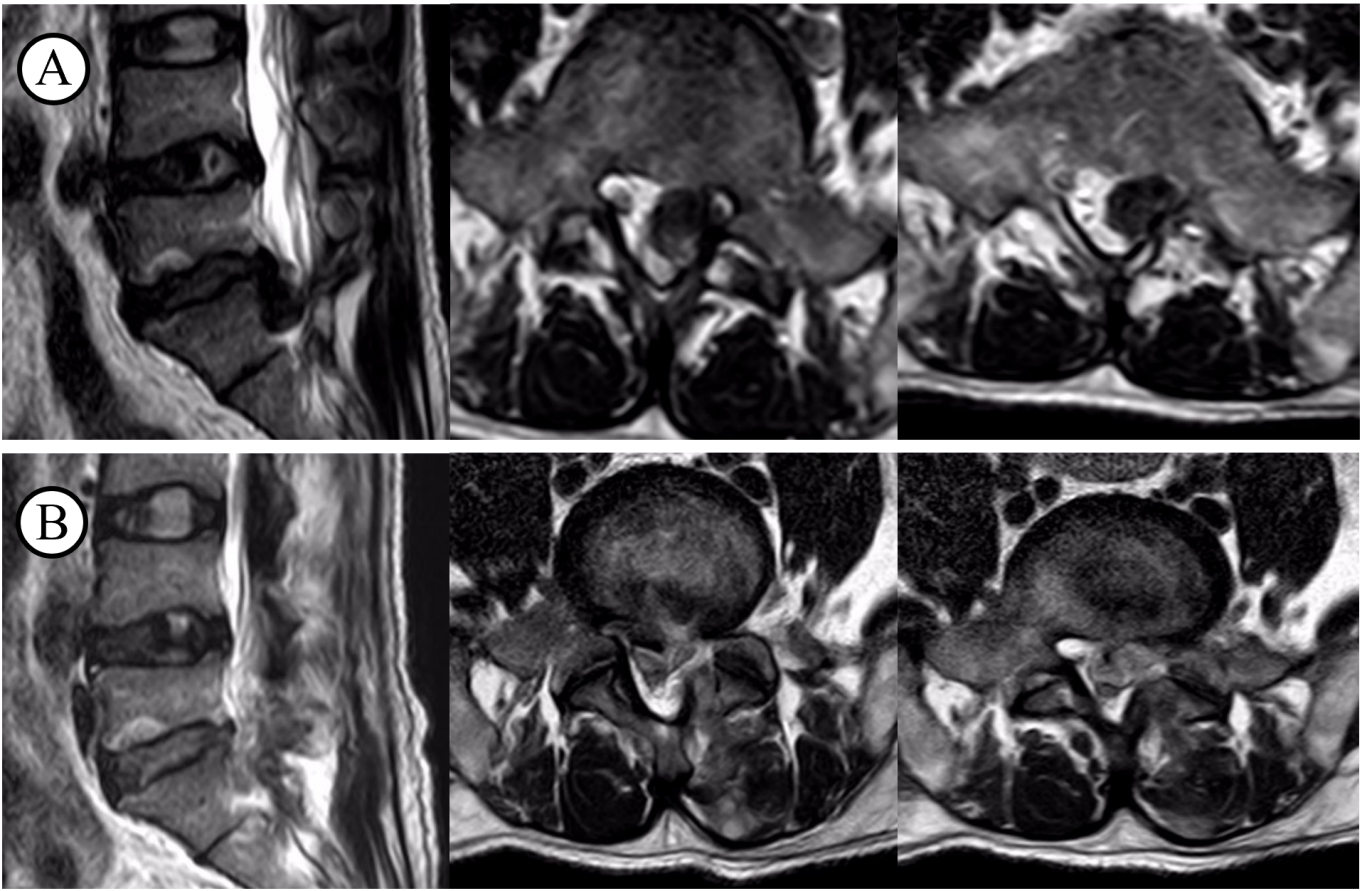
T2WI before and after decompression surgery. **(A)** before the surgery; **(B)** after the surgery.

This project retrospectively analyzed 14 cases (from 7,893 patients treated at the Spine Surgery Department of Affiliated Hospital of Qingdao University [January, 2014 to September, 2024]) of postoperative infections following endoscopic spinal surgery (**Figure 1**). Among the cases analyzed, 9 were caused by Staphylococcus aureus, 1 by Pseudomonas aeruginosa, and 4 had unidentified bacterial infections (blood cultures negative). The patient cohort consisted of 9 males and 5 females, with an average age of 58 years. Most patients had comorbidities, such as type 2 diabetes, hypertension, hypoproteinemia and coronary artery disease. All patients underwent endoscopic spinal surgery for degenerative lumbar disease, with 12 cases involving single-level surgery and 2 involving multilevel surgery. Based on clinical signs and imaging evidence, patients underwent either decompression surgery alone or vertebral fusion and internal fixation implantation surgery (**Table 1**). The reported cases developed symptoms, including limb-related issues, 2 to 17 days postoperatively (average 8.28 days), accompanied by abnormal infection-related test results (**Table 2**).

**Table 1:** General information of 14 cases of endoscopic spine decompression surgery infection.

Case	Sex	Age	Segment	Comorbidity	Surgery Duration (min)	Surgical approach	Implanted internal fixation	Symptom
1	Male	46	L5/S1	——	105	Translaminar approach	No	Low back pain, pain in the left lower limb
2	Female	52	L4/5	——	90	Translaminar approach	No	Low back pain, numbness in the right lower limb
3	Male	64	L1/2	Hypoproteinemia, hypertension, coronary heart disease	100	Transforaminal approach	No	Low back pain
4	Male	47	L4/5	Hypoproteinemia, hypertension	90	Translaminar approach	No	Low back pain, pain in the left lower limb
5	Male	50	L4/5	Type II diabetes, hypertension	85	Translaminar approach	No	Low back pain, pain numbness, decreased sensation and muscle strength in the right lower limb
6	Male	65	L4/5	——	100	Translaminar approach	No	Low back pain
7	Female	68	L2/3, L3/4, L4/5	Type II diabetes	220	Translaminar approach	No	Low back pain, pain in the right lower limb
8	Male	47	L5/S1	Type II diabetes, coronary heart disease	90	Translaminar approach	No	Pain in the left lower limb
9	Female	59	L4/5,L5/S1	Type II diabetes, hypertension	150	Translaminar approach	No	Low back pain
10	Female	76	L4/5	——	120	Transforaminal approach	No	Low back pain, pain and numbness in the right lower limb
11	Male	46	L5/S1	Type II diabetes	100	Transforaminal approach	Yes	Oozing from the wound, pain and numbness in both lower limbs
12	Female	66	L5/S1	Type II diabetes, hypertension	130	Translaminar approach	Yes	Low back pain, pain in both lower limbs
13	Male	61	L4/5	Type II diabetes	75	Translaminar approach	Yes	Low back pain, pain in both lower limbs
14	Male	66	L4/5	hypertension	95	Translaminar approach	Yes	Low back pain, numbness in your left lower limb

**Table 2:** The laboratory test results of 14 cases in this study.

CRP				PCT				ESR			
Pre-surgery	Postoperative infection d1	Postoperative infection d7	Recovery	Pre-surgery	Postoperative infection d1	Postoperative infection d7	Recovery	Pre-surgery	Postoperative infection d1	Postoperative infection d7	Recovery
<0.50	6.45	1.37	<0.50	0.021	0.044	0.04	0.035	4	18	16	12
<0.50	6.73	3.64	<0.50	0.019	0.031	0.032	0.029	6	28	16	10
<0.50	7.02	1.19	<0.50	0.033	0.022	0.014	0.036	4	19	17	13
<0.50	8.41	6.53	<0.50	0.027	0.054	0.027	0.017	5	24	36	16
<0.50	6.89	<0.50	<0.50	0.011	0.017	0.018	0.04	4	33	17	7
<0.50	9.11	4.92	<0.50	0.044	0.045	0.044	0.027	6	21	34	19
<0.50	7.34	7.31	<0.50	0.038	0.076	0.022	0.031	4	25	22	21
<0.50	5.52	2.78	<0.50	0.02	0.027	0.013	0.025	3	26	18	3
0.53	6.21	5.16	<0.50	0.049	0.064	0.031	0.014	7	29	18	15
<0.50	7.65	<0.50	<0.50	0.03	0.059	0.02	0.033	8	38	20	12
<0.50	9.56	8.22	<0.50	0.042	0.078	0.043	0.012	8	37	30	18
<0.50	5.98	9.81	0.51	0.048	0.063	0.039	0.023	12	35	45	28
<0.50	8.78	8.69	<0.50	0.013	0.052	0.025	0.038	10	31	32	30
0.52	9.03	9.12	<0.50	0.029	0.058	0.041	0.022	10	40	39	35

### 2.2 Inclusion criteria

- Endoscopic spinal surgery due to lumbar degenerative changes;
- Postoperative infection symptoms, including rising infection indicators, local redness and swelling, and recurrence of limb symptoms, diagnosed as an infection by a professional physician;
- A follow-up period of at least 12 months.

### 2.3 The course of treatment

**Figure 2:**
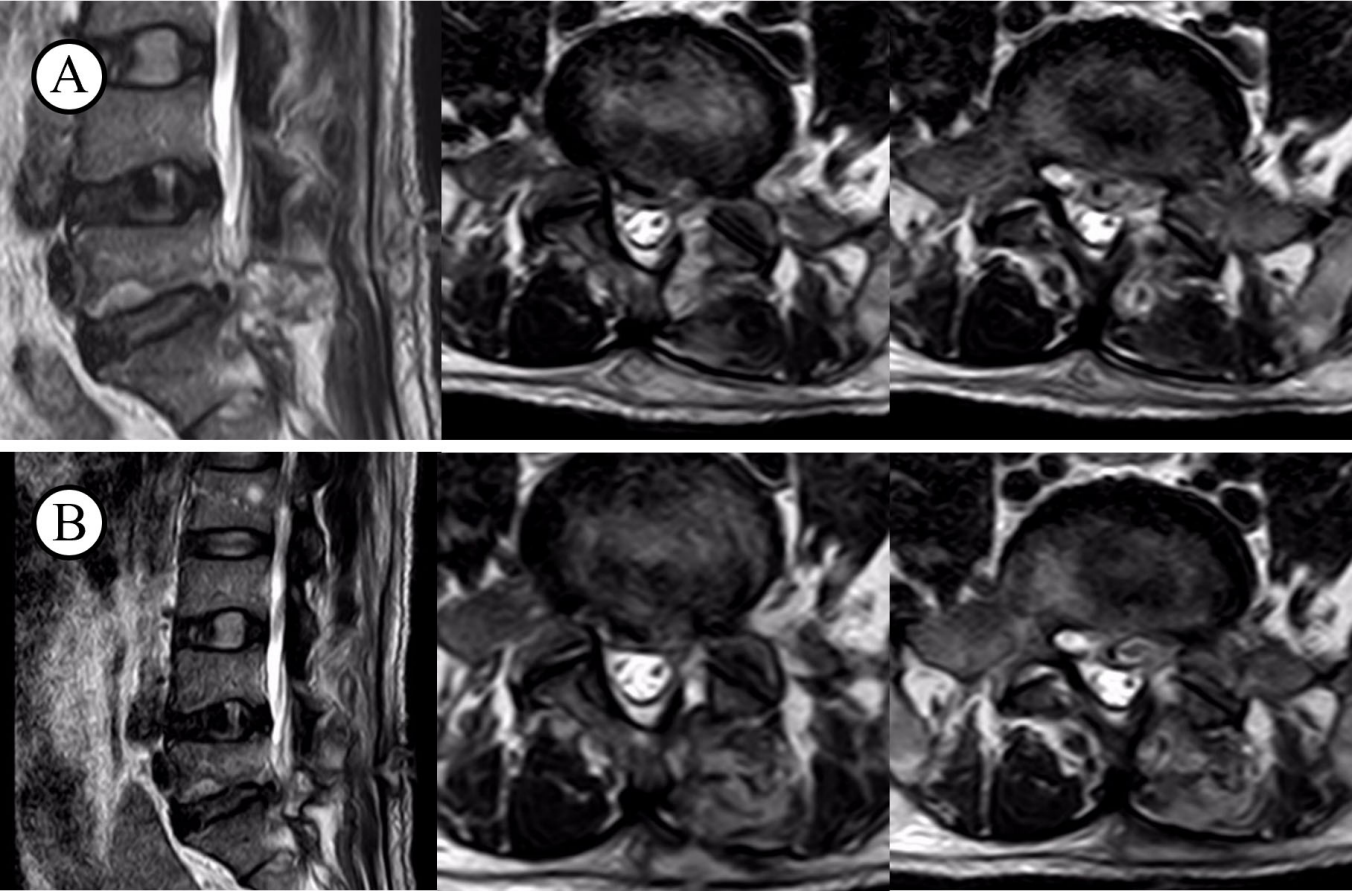
T2WI before and after infection. **(A)** infection on the level of L5S1; **(B)** after recovery from illness.

All patients in the case reports exhibited a recurrence of symptoms, including low back pain and lower limb pain, in the postoperative period. As evidenced by the data presented in **Table 3**, both the Visual Analogue Scale (VAS) and the Oswestry Disability Index (ODI) demonstrated varying degrees of increase, indicating the presence of pathological abnormalities. All patients underwent testing for C-reactive protein (CRP), erythrocyte sedimentation rate (ESR), procalcitonin (PCT), albumin, X-rays, CT, MRI, and blood cultures. On the initial diagnosis of infection in these 14 patients, the following inflammatory markers were observed: The CRP levels were 5.52–9.56 mg/L, with an average of 7.48 mg/L, while the ESR levels were 18–40 mm/h, with an average of 28.9 mm/h. The PCT levels were 0.017–0.078 ng/mL, with an average of 0.049 ng/mL (**Table 2**). An MRI revealed a low T1 signal, high T2 signal, or a combination of both in the intervertebral spaces and adjacent vertebrae at the surgical site (**Figure 2**).

**Table 3:** The treatment and prognosis of 14 cases in this study.

Case		Pathogenic bacteria	SSI diagnosed		Treatment	Follow-up (month)	Last follow-up		
			VAS	ODI			VAS	ODI	MacNab
Decompression Group	1	Staphylococcus aureus	9	49.4%	Sensitive antibiotics: vancomycin and piperacillin sodium for 2 weeks (intravenous infusion), sequential doxycycline and clindamycin for 2 months (oral)	17	0	16.2%	Excellent
	2	Pseudomonas aeruginosa	9	53.7%	Sensitive antibiotics: vancomycin for 5 weeks (intravenous infusion), sequential levofloxacin and cefixime for 3 months (oral)	32	1	12.1%	Excellent
	3	Staphylococcus aureus	7	48.5%	Empiric medication: ceftriaxone for 8 days turn to vancomycin for 4 weeks (intravenous infusion), sequential clindamycin for 3 months (oral)	15	5	40.3%	Fair
	4	Not detected	8	54%	Empiric medication: vancomycin for 6 weeks (intravenous infusion), sequential cefixime for 1 months (oral)	26	0	12.7%	Excellent
	5	Not detected	8	57.6%	Empiric medication: vancomycin for 5 weeks (intravenous infusion)	23	1	23.1%	Good
	6	Staphylococcus aureus	9	65.1%	Posterior lesion clearance + Sensitive antibiotics: vancomycin for 5 weeks (intravenous infusion), sequential cefixime for 3 months (oral)	18	0	15.6%	Excellent
	7	Staphylococcus aureus	8	73.4%	Posterior lesion clearance + Sensitive antibiotics: imipenem for 3 weeks (intravenous infusion), sequential levofloxacin and cefixime 4 months (oral)	24	0	13.7%	Excellent
	8	Staphylococcus aureus	8	69.3%	Empiric medication: vancomycin for 6 weeks (intravenous infusion), sequential cefixime for 3 months (oral)	16	2	25.6%	Good
	9	Staphylococcus epidermidis	8	73.1%	Sensitive antibiotics: vancomycin for 3 months (intravenous infusion), sequential clindamycin for 3 months (oral)	17	3	21.3%	Good
	10	Not detected	8	63.2%	Empiric medication: vancomycin for 5 weeks (intravenous infusion), sequential cefpropone and clindamycin for 2 months (oral)	24	0	15.7%	Excellent
Fusion Group	11	Staphylococcus aureus	8	64%	Sensitive antibiotics: linezolid and levofloxacin for 5 weeks (intravenous infusion), sequential clindamycin and moxifloxacin for 3 months (oral)	21	4	43.5%	Fair
	12	Not detected	8	42.8%	Sensitive antibiotics: vancomycin for 4 weeks (intravenous infusion), sequential clindamycin for 3 months (oral)	33	0	13.2%	Excellent
	13	Staphylococcus aureus	8	74.6%	Empiric medication: vancomycin for 5 weeks (intravenous infusion), sequential clindamycin for 2 months (oral)	18	2	20.1%	Good
	14	Staphylococcus aureus	9	77.5%	Sensitive antibiotics: vancomycin for 4 weeks (intravenous infusion), sequential clindamycin for 3 months (oral)	20	3	22.6%	Good

Bacterial culture results indicated that nine cases were caused by Staphylococcus aureus, one by Pseudomonas aeruginosa, and four had unidentified bacterial infections (blood cultures negative).

Treatment plans were modified in accordance with the results of the cultures. For patients with positive culture results, antibiotics were selected based on sensitivity testing. For patients with negative culture results, empirical broad-spectrum antibiotics were used. The duration of antimicrobial therapy was determined based on regular monitoring of CRP, PCT, ESR levels, and spinal MRI results.

In addition to antibiotic treatment, some patients with identified pathogens through blood culture, who showed a decrease in inflammatory markers after sensitive antibiotic treatment, but with no significant clinical improvement, underwent posterior debridement surgery.

## 3 Results

Following the conclusion of the therapeutic regimen, the final inflammatory markers exhibited a return to normalcy. A short-term follow-up was conducted for all patients following their discharge, with follow-up periods ranging from 15 to 33 months and an average follow-up period of 21.7 months. The final follow-up VAS score averaged 1.5, and the ODI averaged 21.12%. According to the modified MacNab criteria, seven cases were rated as excellent, five as good, and two as fair (**Table 3**). A comparison of the culture results revealed that PCT, CRP, and ESR were elevated in both culture-negative and culture-positive cases (**Table 4**), which has significant implications for the early diagnosis of infection. No significant differences were found in laboratory tests or therapeutic efficacy between culture-negative and culture-positive cases.

**Table 4:** The comparison of cultural result in this study.

Demographic data	Culture Result Negative (n=4)	Culture Result Positive (n=10)	p
Age	59.75 ± 13.67	57.4 ± 8.82	0.705
Sex			0.580
Male	2	7	
Female	2	3	
Symptom			0.315
Lower back pain	0	3	
Lower limb symptoms	0	2	
Lower back pain and limb symptoms	3	5	
Pain combined with motor paresthesia	1	0	
Laboratory result			
ESR	25.00 ± 4.97	30.40 ± 7.47	0.212
PCT	0.039 ± 0.017	0.053 ± 0.019	0.207
CRP	7.43 ± 1.48	7.49 ± 1.30	0.939
Treatment			0.635
Antibiotics alone	4	8	
Drugs combined with surgery	0	2	
MacNab			0.304
Excellent	3	4	
Good	1	4	
Fair	0	2	

### 3.1 Typical case

A 46-year-old male patient was admitted due to lower back pain accompanied by left lower limb numbness and pain for over a month, with symptoms worsening in the past two weeks. The pain was located on the left lateral thigh, and the numbness extended to the left posterior-lateral thigh, left anterior-lateral calf, and left dorsal foot. The patient had no history of hypertension, diabetes, coronary artery disease, or other comorbidities. Physical examination revealed left extensor hallucis longus muscle strength of grade IV, and left straight leg raise at 40° (+). Preoperative laboratory tests showed a white blood cell count of 11.04 × 10^9^/L, CRP < 0.50 mg/L, ESR 4.00 mm/h, and PCT 0.021 ng/mL. Lumbar CT and MRI indicated L5/S1 disc herniation with lumbar spinal stenosis. Based on clinical signs and imaging evidence, the diagnosis of L5/S1 lumbar disc herniation combined with lumbar spinal stenosis was made. After completing preoperative assessments, the patient underwent endoscopic discectomy and spinal decompression surgery. Postoperatively, the limb symptoms alleviated, and laboratory tests returned to normal. The patient was discharged on the third postoperative day.

One week later, the patient was readmitted due to incision redness, swelling, and mild sanguineous drainage, with lower limb pain and numbness relapse. On physical examination, the findings were similar to the initial admission. Laboratory results showed: white blood cells 12.31 × 10^9^/L, CRP 6.45 mg/L, PCT 0.044 ng/mL, ESR 18.00 mm/h. Blood cultures identified Staphylococcus aureus infection. Based on empirical therapy and culture results, intravenous vancomycin and piperacillin sodium were administered for 2 weeks. After 2 weeks, infection markers normalized, limb symptoms alleviated, and the patient was discharged with instructions for sequential oral antibiotics for 2 months. Follow-up at 17 months showed a final VAS of 0, ODI of 16.2%, and a modified MacNab score of Excellent (Table 1-1, 3-1).

## 4 Discussion

Lumbar intervertebral space infection is not common in clinical practice and can be divided into primary and secondary infections. Infections that occur after surgery are called secondary intervertebral infections, and this complication has always been a major concern for spine surgeons (Babic and Simpfendorfer, 2017; Dowdell et al., 2018). Most surgical site infections in patients after spinal endoscopy are intervertebral space infections, and infections at the surgical site of spinal endoscopy can directly affect the postoperative recovery of patients and the therapeutic effect of the disease, so their occurrence has caused widespread concern (Dai et al., 2020; Tsantes et al., 2020). Due to the deep location of spinal endoscopic surgery and the complex surrounding structures, there are some difficulties in diagnosis and treatment. Early symptoms are often mistaken for poor postoperative results or temporary exacerbations, which poses a challenge for clinicians to confirm the diagnosis (Schimmel et al., 2010). In addition, some patients do not have a significant increase in body temperature, which makes them more likely to be overlooked. In our cases or cases in the literature, the most common clinical manifestations of post-spinal endoscopic infections are focal spinal pain, neurological deficits, and fever (Nasser et al., 2010; King et al., 2020). This is the typical diagnosis. However, in our statistical cases, these characteristics are not typical and have poor specificity, which also confuses clinicians in treatment, resulting in prolonged treatment and delayed diagnosis and treatment in many patients with post-spinal endoscopic infection in the study. For patients with back pain and fever after spinal endoscopy, it is important to reasonably consider post-operative infection before the onset of neurological deficits. We must respond actively to avoid serious complications. At present, there is little relevant literature reporting the risk factors for infection after spinal endoscopic surgery. Therefore, it is necessary to comprehensively analyze the relevant risk factors in existing cases of infection after spinal endoscopy, and take corresponding interventions for early diagnosis, treatment, and prevention to avoid this complication.

Spinal intervertebral space infection usually lacks obvious clinical symptoms, with low back pain and surgical incision pain as the most common early manifestations. Other clinical features such as incision redness and swelling, fever, local tenderness, and a small amount of purulent discharge are rare, and there is a lack of specific signs and imaging manifestations, which further increases the difficulty of early diagnosis of the disease (Tsantes et al., 2020). Staphylococcus aureus is the most common pathogen causing spinal intervertebral space infection (Abdul-Jabbar et al., 2013; Salaffi et al., 2021). Although most patients will have some abnormal increases in laboratory test indicators, such as white blood cell count, CRP and ESR, in the early stages of spinal intervertebral space infection, these elevated indicators are not specific and cannot be used to confirm the diagnosis of spinal intervertebral space infection, so there are certain difficulties in the early diagnosis of the disease (Kang et al., 2010). X-rays and CT are convenient and fast methods that can better describe the degree of bone involvement, but they are not as good as MRI in diagnosing infection after spinal endoscopy. Spinal MRI is the first choice for diagnosing spinal intervertebral space infection (Stäbler and Reiser, 2001; Salaffi et al., 2021). When a patient presents with back pain, neurological deficits, or fever, and hematological tests show elevated CRP and ESR levels, an early spinal MRI should be performed as soon as possible. MRI has high accuracy, sensitivity, and specificity in the diagnosis of early-stage interspinous space infection. Long T1 hypointense signals in the adjacent vertebral body of the intervertebral disc after MRI scanning can play a key role in determining whether there is an early intervertebral space infection. This investigation mainly screened relevant cases based on the results of MRI examinations, and combined them with evidence related to back pain, severe pain at the surgical incision site, and laboratory test results such as elevated white blood cell count, ESR, and CRP, to finally confirm 14 cases of infection.

Intraoperative continuous irrigation during spinal endoscopy can reduce surgical trauma and blood loss, as well as lower the incidence of postoperative intervertebral space infections (Watanabe et al., 2010). According to the literature, factors such as diabetes, alcohol abuse, smoking history, obesity, hypoproteinemia, and advanced age are associated with postoperative intervertebral space infections (Mehta et al., 2013; Martin et al., 2016). Additionally, infections after spinal endoscopy may be related to the learning curve of the endoscopic surgeon, the surgical segment, and the surgical approach. Repeated punctures and fluoroscopy during the procedure can lead to instrument contamination, and prolonged surgery times increase the risk of infection. A study by Ogihara et al. suggests that a surgical duration exceeding two hours is a significant predictive factor for postoperative intervertebral space infections (Ogihara et al., 2021). As surgery time increases, the risk of instrument contamination and bacterial spread in the operative field also rises, thus elevating the likelihood of infection.

Currently, there are limited reports on the clinical characteristics of postoperative intervertebral space infections following spinal endoscopy, and early diagnosis is challenging, often being confused with common postoperative discomforts. In this study, we summarize the unique clinical features of postoperative intervertebral space infections following spinal endoscopy based on our case series: 1) In contrast to traditional open surgeries, where infection typically affects the vertebral body and soft tissues, the infections in our cases were confined to the treated intervertebral spaces, likely due to repeated punctures and instrument contamination during surgery; 2) postoperative intervertebral space infections following spinal endoscopy develop rapidly, with the time between the initial surgery and the onset of postoperative intervertebral space infections ranging from 2 to 17 days (average 8.28 days), predominantly presenting as early infections (≤30 days); 3) Clinically, patients primarily experienced more severe low back or lower limb pain, often accompanied by fever. Due to the rapid progression of postoperative intervertebral space infections following spinal endoscopy, their symptoms and signs lack specificity, making early diagnosis crucial.

MRI is the imaging method of choice and can accurately demonstrate changes in the vertebral body, margins, intervertebral spaces, and soft tissues at different stages, providing a clear view of the affected spinal canal structures. This aids in assessing the extent of infection and formulating individualized treatment plans. Typical MRI findings include low signal intensity on T1-weighted images and high or mixed signal intensity on T2-weighted images of the infected intervertebral space and adjacent vertebrae, with signs of swelling or thickening in the nearby soft tissues. Localized enhancement may be visible on contrast-enhanced images (Stäbler and Reiser, 2001; Salaffi et al., 2021). However, MRI as the sole diagnostic tool for early postoperative intervertebral space infections is not entirely reliable and must be supplemented by abnormal biochemical markers for accurate diagnosis. In all of our cases, MRI showed typical infection signals in the intervertebral space, with elevated CRP and ESR levels, and some cases had an increased WBC. Thus, we hypothesize that the key to early postoperative intervertebral space infections identification is the presence of severe low back or radicular pain within two weeks post-surgery, combined with typical MRI findings and elevated inflammatory markers.

Although no significant differences were found between patients with positive and negative blood cultures regarding general condition, diagnosis, treatment, or prognosis in our case series, the small sample size limits the accuracy of our conclusions. Empirical antibiotic therapy remains challenging before the pathogen is identified or when bacterial cultures are negative. Empirical antimicrobial agents should cover Gram-negative bacteria and methicillin-resistant Staphylococcus aureus (MRSA), including clindamycin, vancomycin, flucloxacillin, cefepime, ciprofloxacin, and ceftriaxone (Tsiodras and Falagas, 2006; Palmowski et al., 2020). For culture-negative or biopsy-negative cases, third-generation cephalosporins or fluoroquinolones combined with clindamycin or vancomycin may be considered. The optimal duration of antibiotic therapy is still debated. Treatment duration can range from 4 to 12 weeks, but a sufficient antibiotic course is crucial for successful infection control. It is generally recommended to administer intravenous antibiotics for 6 weeks, followed by oral antibiotics for 6 weeks (Quiñones-Hinojosa et al., 2004; Dubée et al., 2012; Benavent et al., 2021). Changes in inflammatory markers can indicate the effectiveness of the antimicrobial therapy, with CRP being more significant than ESR. A 50% reduction in CRP levels per week is considered an indicator of effective treatment (Khan et al., 2006; Kang et al., 2010). We believe that using an adequate and reasonable antibiotic regimen is key to successful postoperative intervertebral space infection treatment. It is advised to administer intravenous antibiotics for at least 4 weeks, and if inflammatory markers return to or near normal levels upon follow-up, a switch to oral antibiotics for 8 to 12 weeks can be considered. Once inflammatory markers normalize, MRI shows a return to normal or near-normal T1 signal and mixed T2 signal in the affected areas, and CT reveals bone sclerosis in the vertebral body and adjacent bone fusion or bone bridge formation between vertebrae, antibiotic therapy can be discontinued (Tsantes et al., 2020).

When there is no neurological deficit or only mild neurological impairment, conservative treatment can be initially pursued. If conservative treatment is ineffective or the condition worsens, surgical intervention may be required (Lener et al., 2018). Conservative treatment is appropriate for cases without nerve compression or mechanical instability; however, for patients with nerve damage, severe vertebral destruction resulting in poor spinal stability, or those who do not respond to antibiotic treatment, debridement combined with internal fixation surgery should be performed. Percutaneous endoscopic surgery allows direct microscopic visualization of the lesion, enabling its removal and the collection of tissue samples for pathogen culture (Tsantes et al., 2020). Continuous irrigation and drainage after catheterization help effectively clear inflammatory tissue, and this method has been successfully applied in the treatment of spinal infections and postoperative intervertebral space infections. However, the long-term curative effect of this approach still requires further validation. Despite surgical intervention and extended intravenous antibiotic therapy, the neurological prognosis remains a critical concern (Stüer et al., 2013). The goal of surgery should be appropriate neurological decompression, control of the infection source, and restoration of spinal stability.

Prevention is paramount when it comes to infection. Preventive measures include the rational use of antibiotics, strict adherence to aseptic techniques, proper instrument sterilization, attention to puncture angle, direction, and channel establishment, and minimizing the surgical exposure time. Additionally, preoperative assessments should include white blood cell count, ESR, CRP levels, and chest X-rays. If signs of infection are suspected preoperatively, further antimicrobial treatment must be administered before proceeding with surgery (Anderson et al., 2017; Spina et al., 2018; Aleem et al., 2020).

In conclusion, although postoperative intervertebral space infections following spinal endoscopy are rare, they can be severe and therefore require early diagnosis, treatment, and prevention. We recommend the following measures to reduce the occurrence of postoperative intervertebral space infections: 1) Actively manage underlying conditions during the perioperative period while ensuring proper preparation of the surgical site; 2) Plan the puncture route in advance to reduce the number of punctures and fluoroscopy during surgery; 3) Maintain strict sterility throughout the procedure, avoid contamination of surgical instruments during fluoroscopy, and minimize the number of instrument passages through the surgical channel; 4) Ensure that the surgical team is proficient in technique and aim to minimize overall surgery time.

## 5 Conclusion

Postoperative lumbar intervertebral space infections following spinal endoscopy are uncommon but present significant challenges when they occur. Our retrospective analysis identified that while the overall incidence remains low, infections predominantly developed within the first two weeks postoperatively. The most common clinical manifestations—localized back pain, neurological deficits, and fever—lacked specificity, complicating early diagnosis. MRI, in conjunction with elevated inflammatory markers such as CRP and ESR, proved crucial for timely detection.

No significant correlation was found between the presence of positive or negative blood cultures and clinical outcomes. While empirical antibiotic therapy was effective in most cases, early identification of the causative pathogen remains important for optimizing treatment strategies.

Strict aseptic techniques, careful preoperative planning, and minimizing intraoperative contamination are essential preventive measures. Given the inherent limitations of this retrospective study, including a small sample size, further prospective, multicenter, randomized controlled trials are necessary to clarify risk factors and establish more precise guidelines for prevention and management.

## Data Availability

The original contributions presented in the study are included in the article/supplementary material. Further inquiries can be directed to the corresponding author/s.
